# Evaluation of the reasons for the non‑COVID‑19 status: A socio‑demographic analysis

**DOI:** 10.3892/mi.2023.127

**Published:** 2023-12-05

**Authors:** Onur Öztürk, Alaıddın Domaç, Șuayıp Ceylan, Arzu Ayraler, Mehmet Akif Tapur, Muhammet Ali Oruç

**Affiliations:** 1 Department of Family Medicine, Faculty of Medicine, Samsun University, Samsun 55070, Turkey; 2 Clinic of Anesthesiology and Reanimation, Bafra State Hospital, Samsun 55400, Turkey; 3 Department of Family Medicine, Faculty of Medicine, Giresun University, Giresun 28100, Turkey; 4 Vezirköprü District Health Directorate, Samsun 55900, Turkey

**Keywords:** coronavirus disease 2019, socio-demographic factors, disease

## Abstract

The present study aimed to evaluate the reasons behind the fact that some individuals did not contract coronavirus disease 2019 (COVID-19), considering certain socio-demographic data. The present cross-sectional study was conducted at a state hospital between February 1, 2022 and March 1, 2022. The study group consisted of individuals who never had COVID-19, and the control group consisted of individuals who did not know at the time of the study whether they had COVID-19. A data collection form consisting of 29 questions created based on a literature review was used. A total of 2,958 subjects (study group, 669; control group, 2,289) were included; of these, 53.1% were females and 46.9% were males. It was found that housewives (P<0.001), individuals with secondary school and lower education levels (P=0.02), those residing in rural areas (P=0.003), those who received a combination vaccine (P<0.001), those with chronic diseases (P=0.016), those who consumed more fruits (P=0.001), those who used N95 masks (P=0.002), those with pets (P<0.001) and those who did not follow the news regarding COVID-19 (P=0.016) had a higher probability of not contracting COVID-19. On the whole, the present study observed that socio-demographic factors affected the non-COVID-19 status.

## Introduction

The fight against the coronavirus disease 2019 (COVID-19) pandemic continues worldwide. Hundreds of thousands of cases are reported each day, with thousands of individuals succumbing to the disease due to the fact that vaccination, treatment and preventative approaches have not yet been completely successful. The clinical presentation of COVID-19 in adults ranges from an asymptomatic infection to severe pneumonia, which may be associated with multi-organ failure (1).

In addition to the recommendations of the World Health Organization (WHO), each country develops its own policies and tries to take the appropriate action. For example, urging individuals to stay at home is an effective approach to prevent the spread of COVID-19. The protective effects of such measures on controlling the spread of COVID-19 and effectively avoiding the overburdening of healthcare systems during the pandemic are well known. These social distancing measures have been adopted by governments worldwide. However, compliance with the ‘stay at home’ recommendation varied according to country (2). Stay-at-home policies are categorized as follows: 0, no measures; 1, recommendations to not leave the house; 2, required to not leave the house with exceptions for daily exercise, grocery shopping, and ‘essential’ trips; 3, required to not leave the house with minimal exceptions (for example, allowed to leave only once a week, or only one person can leave at a time) (https://www.unicef.org/turkiye/en/press-releases/least-1-7-children-and-young-people-has-lived-under-stay-home-policies-most-last).

In addition to prevention efforts, individual characteristics and medical histories of individuals are known to be the key factors affecting the transmission of COVID-19. Emerging variants of COVID-19 are continually creating high levels of global public health concerns and panic (3). Furthermore, COVID-19 continues to have major health, economic and social consequences worldwide according to the WHO International Health Regulations Emergency Committee (4).

Despite the high risk of COVID-19 transmission, not becoming infected with COVID-19 has been considered a virtue, and the characteristics of individuals who do not become infected with COVID-19 have become the matter of discussion. However, this topic has not been adequately investigated and may pave the way for the development of interesting guidelines. The present study aimed to examine the reasons behind the fact that some individuals do not contract COVID-19, considering certain socio-demographic data.

## Subjects and methods

### Study design

The present cross-sectional study was conducted at Bafra State Hospital, Samsun, Turkey between February 1, 2022 and March 1, 2022. Patients from the COVID-19 outpatient and pediatric clinics of the health institution were excluded from the study. To reach the participants among all applications to the hospital, two teams comprising four volunteer pre-determined healthcare professionals conducted daily interviews. In the daily triage unit of the hospital, emergency triage unit and intensive care clinics, these professionals routinely conducted pre-assessments of patients for the presence of COVID-19. No additional fees were paid to the teams. Participants were selected by simple randomization as this ensures that the assignment of a subject to a particular group is completely random. The first team asked the participants whether they had been previously infected with COVID-19. The team then made the data collection form available to those who had not previously contracted COVID-19, and based on the collected data, these individuals were included in the study group. The second team provided the form to all participants, and based on the collected data, these individuals were included in the control group. Notably, the control group was considered as the average population; they may or may not have had COVID-19.

### Study participants

The inclusion criterion was the age of ≥18 years. The exclusion criteria were the age of <18 years, a diagnosis of psychotic illness and the presence of any disease preventing communication.

### Data collection tools

The data collection form was created according to a literature review (1,2,4) and consisted of 29 open-ended questions examining socio-demographic data and habits (nutrition, news following, pet adoption, smoking and alcohol consumption, sleep duration). The form took an average of 10 min to complete, and there were no repeat participant interviews.

### Statistical analysis

Data analysis was conducted using SPSS 22.0 for Windows (IBM Corp.). Descriptive criteria are presented as the mean, standard deviation and percentage distribution. The conformity of the data to a normal distribution was evaluated using the Kolmogorov–Smirnov test. The unpaired Student's t-test was used to compare continuous variables between the two groups, and the Chi-squared test was used to compare distributions. Logistic regression analysis was performed to examine the combined effects of independent variables found to have a statistically significant effect on the absence of COVID-19 in individual analyses. A P-value <0.05 was considered to indicate a statistically significant difference.

### Ethics approval

The present study was conducted with the permission of the Turkish Ministry of Health, and ethical approval was obtained from the Samsun Education and Research Hospital Ethics Committee (protocol no. BAEK/2022/1/14). The study followed the Declaration of Helsinki, and written informed consent was obtained from all subjects.

## Results

Of the 2,958 subjects (study group, 669; control group, 2,289) who participated in the present study, 53.1% were females and 46.9% were males. The mean age of the participants was 38.1±17.2 years. A comparison between the two groups in terms of various characteristics and habits is presented in Tables I and II. As regards statistical analyses, no statistically significant differences were found between the two groups in terms of sex and age. However, it was found that housewives were more likely to not contract COVID-19 (P<0.001). Moreover, individuals with secondary school and lower education levels were more likely to not contract COVID-19 than those with high school and higher education levels (P=0.020). Based on the analysis of residential location, the probability of not contracting COVID-19 was higher among subjects who resided in rural areas (P=0.003). When the participants who received the Sinovac or Biontech vaccine were compared according to their COVID-19 status, no statistically significant difference was found between those who received the Sinovac vaccine and those who received the Biontech vaccine. However, when the combination vaccine was included in the analysis, those who received a combination vaccine were more likely to be COVID-19-negative than those who received a single vaccine (P<0.001). Based on the within-group comparison of participants with chronic diseases, the number of subjects not contracting COVID-19 was higher among those with chronic diseases (P=0.016) (Table I).

It was also observed that those who consumed more fruits were less likely to contract COVID-19 (P=0.001). When the subjects were compared based on the use of protection methods, those using N95 masks had a statistically significantly lower rate of contracting COVID-19 than those using other protection methods (P=0.002). Additionally, it was found that subjects who kept animals indoors or outdoors had a higher likelihood of not contracting COVID-19 (P<0.001). Moreover, subjects who did not follow the news regarding COVID-19 had a higher probability of not contracting COVID-19 (P=0.016). There were no statistically significant differences between the groups in terms of other characteristics (Table II).

The effects of the independent variables (profession, daily time spent outside the home, fruit consumption, pet adoption, vaccine type) on the dependent variable (non-COVID-19 status of the study participants) were then examined (Table III) using logistic regression analysis (backward logistic regression). Considering unemployment as a reference in terms of occupation, the probability of not contracting COVID-19 was 2.6-fold higher in workers, 1.4-fold higher in students, 1.5-fold higher in housewives and 1.7-fold higher in self-employed individuals. When Sinovac was used as a reference in terms of the vaccination status, it was found that those who received a combination vaccine were 1.4-fold more likely to not contract COVID-19. When eating three or less servings of fruits was considered a reference, those who consumed four or more servings of fruits were 1.5-fold more likely to not contract COVID-19, and those who kept animals outside were 1.6-fold more likely to not contract COVID-19.

## Discussion

The literature regarding COVID-19 has been constantly expanding. However, the reasons behind why certain individuals do not contract the infection remain unclear. The present study aimed to explore these reasons using certain demographic data and habits. The demographic data of the average population were compared with the cohort of individuals who did not contract COVID-19. The present study has potential value in terms of new pandemics that may develop with other strains of the virus. At this point, one of the best examples of this is the flu. The influenza A (H1N1) subtype manifested itself as Spanish flu and swine flu, H2N2 as Asian flu and H3N2 as Hong Kong flu. Common measures to be taken can be effective in protecting against different strains (5).

Some studies conducted in Turkey have reported that the majority of patients with COVID-19 are males and of an older age. Healthcare workers are also at a higher risk of contracting COVID-19 (6,7). This result has been attributed to the fact that ACE2 receptor expression is high in males; moreover, males lack estrogen and chromosome X protection (8). The progression of the disease becomes more severe with age due to the increase in the number of concomitant diseases. In addition, the increased number of fat cells in obese individuals provides more entry points for the virus (9). In the present study, housewives were found to be less likely to contract COVID-19, although the study did not identify a significant difference between the groups in terms of sex, age, body mass index, the number of children living together or marital status. The fact that unemployed subjects spent less time at home during the pandemic period, when the economy declined, as with every other parity, may have exacerbated the risk of disease transmission. Moreover, it was observed that individuals with lower education levels were more unlikely to be infected by COVID-19. This may be due to the fact that housewives and students generally stayed at home during the pandemic. The fact that these individuals mainly resided in rural areas can also be considered as a contributing factor. In fact, living in the countryside has been found to reduce risk, and those who live in rural areas may receive benefits due to the natural environment that has less human density.

The first study examining the association between COVID-19 and blood groups in Turkey reported an increased COVID-19 prevalence in individuals with blood group A and a decreased prevalence in those with blood groups B, AB and particularly, O (10). However, there was no significant difference in terms of the ABO blood group system when compared with healthy individuals. In terms of the Rh blood group system, Rh positivity was found to be markedly higher in patients diagnosed with COVID-19, and the Rh(-) blood group was found to be a protective factor, whereas the Rh(+) blood group was a predisposing factor (10). According to the study by Ray *et al* (11), blood type O may be associated with a lower risk of severe COVID-19 illness or related mortality. However, the present study reported that blood type had no impact on the absence of COVID-19. The socio-biological infrastructure of the geography where the research was conducted may have affected this result.

A number of comorbid conditions have been found to be associated with the clinical course, severity and mortality related to COVID-19. Among these comorbidities, the most common chronic systemic diseases are hypertension, diabetes mellitus, coronary artery diseases and cancer (12-14). In the present study, it was found that individuals with comorbid conditions were more likely to not contract COVID-19. This result is inconsistent with the relevant literature (12-14). In addition, it was observed that chronic drug use had no effect on the non-COVID-19 status. The fact that existing comorbidities in drug users tend to be under control may explain this result. One of the possible explanations is that these individuals are particularly sensitive to the transmission of the disease due to factors, such as staying at home, hygiene, fear of death, etc.

It has been suggested that an increased alcohol consumption weakens long-term acquired immunity (15). Some studies have reported that cigarette use increases COVID-19-related morbidity and mortality rates (16,17). However, in the present study, alcohol and cigarette consumption had no marked effect on the non-COVID-19 status. Studies regarding the number and quality of the effect of alcohol consumption on COVID-19 are limited in the literature (18,19).

The study by Çerçi *et al* (20) found that those who wished to remain updated about COVID-19 most often prefer television as a tool, and the majority of social media users use social media to receive updates about the COVID-19 crisis. The present study reported that not following the news is effective in avoiding the disease. It was believed that the use of media had a disturbing effect and thus, negatively affected the immune system.

Chronic poor sleep is a causal risk factor of respiratory infections and contributes to the severity of these infections. This underscores the role of sleep in maintaining an adequate immune response against pathogens. According to the findings of the present study however, sleep had no significant impact on this process. The decrease in the need for sleep due to the fact that individuals who do not leave their homes during the pandemic period do not spend enough energy may also be effective in this regard. However, as the COVID-19 pandemic has led to an increase in the number of individuals suffering from insomnia, safety interventions, such as sleep management and treating individuals with insomnia, can be promoted to reduce infections and save lives (21).

Consuming large amounts of fruit and vegetables has been reported as a risk-associated behavior (22). However, the decline in the consumption of these foods during the pandemic has not gone unnoticed (23). In a study on the dietary habits of individuals in the Thrace region during the COVID-19 pandemic, it was observed that those infected with COVID-19 consumed more water (24). The data of the present study indicated that higher numbers of fruit servings favored the prevention of the disease. However, no significant association was found with the amount of water intake. Additionally, the use of dietary supplements has not been reported as an effective approach. It can thus be assumed that the minerals and vitamins ingested with the consumption of fruits may provide effective protection, whereas the calories ingested while overeating and insulin secretion can increase susceptibility to the disease.

Vaccination reduces mortality and morbidity due to COVID-19 and protects public health (25). According to the data presented herein, a combination vaccine was beneficial for COVID-19, regardless of the type and number of vaccines. There are insufficient academic data for the Sinovac/Biontech/Turkovac combinations administered in Turkey; however, data from completed and ongoing studies provide evidence that different combinations of vaccines enhance the immune response. For example, three separate studies reported that the combination of the Oxford/AstraZeneca vaccine with mRNA vaccines elicited a stronger immune response than individual vaccines (26-28).

The study by Demir and Apaydın (29) found that those who had pet cats and dogs were less likely to contract COVID-19 than those who did not, although this difference was not statistically significant. In the present study, it was found that keeping animals at home or outside the home increased the likelihood of not contracting the disease. This difference may be attributed to previous susceptibility to other coronavirus groups in pets.

Following hand hygiene and social distancing, masks are the most effective personal protective equipment (30). It has been observed that the use of N95 masks is more directly proportional to not becoming infected than using other forms of protection.

The present study has certain limitations which should be mentioned. First, the present study was a single-center study; therefore, the variables that show or do not show significant differences may differ if a multicenter study was conducted. Second, the present study was based on individual statements; thus, it affects the objectivity. Finally, no valid and reliable questionnaire was designed, and this reduces the analytical power of the study.

In conclusion, among the evaluated parameters, being a housewife or self-employed, having a low level of education, residing in the countryside, being vaccinated with different types of vaccines, having a chronic illness, consuming more fruits, being protected with N95 masks, keeping animals at home or outside, and not following the news were found to be positively associated with the non-COVID-19 status.

## Figures and Tables

**Table I:** Comparison of the study groups in terms of socio-demographic characteristics.

Characteristic	Case (n=669)	Control (n=2,289)	P-value	Cramer's V/Cohen's d
Sex, n (%)			0.626	
Female	350 (52.3%)	1,222 (53.4%)		
Male	319 (47.7%)	1,067 (46.6%)		
Age (years), mean ± SD	39.1±17.7	37.8±17.0	0.073	
Body mass index (kg/m^2^), mean ± SD	25.3±4.8	24.9±5.1	0.122	
No. of children living with subjects, mean ± SD	1.2±1.3	1.1±1.4	0.356	
Profession, n (%)			**0.001**	0.063
Unemployed	69 (10.3%)	344 (15%)		
Healthcare personnel	30 (4.5%)	135 (5.9%)		
Civil servant	32 (4.8%)	201 (8.8%)		
Worker	27 (4%)	59 (2.6%)		
Student	139 (20.8%)	495 (21.6%)		
Housewife	193 (28.8%)	548 (23.9%)		
Self-employed	179 (26.8%)	507 (22.1%)		
Educational status, n (%)			**0.020**	0.051
Middle school and lower	275 (41.1%)	828 (36.2%)		
High school and higher	394 (58.9%)	1,461 (63.8%)		
Marital status, n (%)			0.147	
Single	228 (34.1%)	873 (38.1%)		
Married	427 (63.8%)	1,365 (59.6)		
Divorced/widowed	14 (2.1%)	51 (2.2%)		
Blood group, n (%)			0.744	
A Rh(+)	323 (48.3%)	1,075 (47%)		
A Rh(-)	25 (3.7%)	111 (4.8%)		
B Rh(+)	61 (9.1%)	240 (10.5%)		
B Rh(-)	20 (3%)	74 (3.2%)		
0 Rh(+)	122 (18.2%)	432 (18.9%)		
0 Rh(-)	45 (6.7%)	129 (5.6%)		
AB Rh(+)	59 (8.8%)	187 (8.2%)		
AB Rh(-)	14 (2.1%)	41 (1.8%)		
Place of residence, n (%)			**0.003**	0.037
Urban	466 (69.7%)	1,724 (75.3%)		
Rural	203 (30.3%)	565 (24.7%)		
No. of doses of vaccine, mean ± SD	2.5±1	2.5±0.9	0.536	
How many doses of vaccine received, n (%)			0.794	
0	45 (6.7%)	145 (6.3%)		
1	23 (3.4%)	69 (3%)		
≥2	601 (89.8%)	2,075 (90.7%)		
Type of vaccine administered, n (%)			**0.001**	0.114
Biontech	406 (60.7%)	1,566 (68.4%)		
Sinovac	105 (15.7%)	346 (15.1%)		
Turkovac	2 (0.3%)	2 (0.1%)		
Combination	111 (16.6%)	230 (10.1%)		
Unvaccinated	45 (6.7%)	145 (6.3%)		
Presence of chronic diseases, n (%)			**0.016**	0.043
No	541 (80.9%)	1,922 (84%)		
Yes	128 (19.1%)	367 (16%)		
Chronic drug use, n (%)			0.506	
No	584 (87.3%)	2,014 (87.9%)		
Yes	85 (12.7%)	275 (12.1%)		

Cramer's V/Cohen's indicates the effect size. Values in bold font indicate statistically significant differences (P<0.05).

**Table II:** Comparison of the groups in terms of habits.

Habit	Case (n=669)	Control (n=2,289)	P-value	Cramer's V/Cohen's d
Smoking, n (%)			0.600	
Yes	263 (39.3%)	912 (39.8%)		
No	406 (60.7%)	1377 (60.2%)		
Alcohol, n (%)			0.694	
Yes	61 (9.1%)	219 (9.6%)		
No	608 (90.9%)	2,070 (90.4%)		
No. of meals, n (%)			0.180	
≤2	181 (27.1%)	518 (22.6%)		
≥3	488 (72.9%)	1,771 (77.4%)		
No. of servings of fruits, n (%)			**0.001**	0.065
≤3	565 (84.5%)	2,073 (90.6%)		
≥4	104 (15.5%)	216 (9.4%)		
Daily amount of sleep (hours), mean ± SD	7.8±1.6	7.7±1.7	0.264	
Daily water consumption (liters), mean ± SD	1.87±0.9	1.95±0.9	0.063	
Protection method used, n (%)			**0.002**	0.068
Mask	594 (88.8%)	1,955 (85.4%)		
Double mask	52 (7.8%)	232(10.1%)		
N95	12 (1.8%)	15 (0.7%)		
N95 + mask	11 (1.6%)	73 (3.2%)		
No method	0 (0%)	14 (0.6%)		
Dietary supplement for protection, n (%)			0.694	
No	628 (93.9%)	2,139 (93.4%)		
Yes	41 (6.1%)	150 (6.6%)		
Presence of pets at home, n (%)			**0.001**	0.016
No	551 (82.4%)	1,963 (85.8%)		
Yes	118 (17.6%)	326 (14.2%)		
Keeping animals outside, n (%)			**0.001**	0.061
No	478 (71.4%)	1,909 (83.4%)		
Yes	191 (28.6)	380 (16.6%)		
Type of heating used, n (%)			0.459	
Natural gas	444 (66.3%)	1,528 (66.8%)		
Stove + fireplace	225 (33.7%)	761 (33.2%)		
Following the news on COVID-19, n (%)			**0.016**	0.061
Yes	547 (81.8%)	1,959 (85.6%)		
No	122 (18.2)	330 (14.4%)		

Cramer's V/Cohen's d indicates the effect size. Values in bold font indicate statistically significant differences (P<0.05).

**Table III:** Analysis of the effects of various characteristics on the non-COVID-19 status in the study groups using logistic regression analysis.

Independent variables	B	S.E.	Wald	SD	Exp (B)
Profession					
Unemployed^a^					
Healthcare personnel	0.185	0.260	0.507	0.476	1.203
Civil servant	-0.101	0.247	0.167	0.682	0.904
Worker	0.966	0.290	11.134	0.001	**2.628**
Student	0.363	0.180	4.040	0.044	**1.437**
Housewife	0.415	0.170	5.955	0.015	**1.514**
Self-employed	0.537	0.171	9.877	0.002	**1.711**
Vaccine type					
Sinovac^a^					
Biontech	-0.110	0.131	0.705	0.401	0.896
Combination	0.351	0.170	4.262	0.039	**1.420**
Daily time spent outside the home	-0.024	0.013	3.304	0.069	0.976
No. of fruits consumed					
≤3^a^					
≥4	0.396	0.141	7.882	0.005	**1.486**
Keeping animals outside	0.471	0.113	17.501	0.001	**1.602**

Nagelkerke R^2^=0.124; Omnibus Chi-squared=233.8; P=0.001; Hosmer and Lemeshov=14.5. Dependent variable, whether subjects have COVID-19 or not. Values in bold font indicate statistically significant differences (P<0.05).

^a^Variable based on regression analysis within the group.

## Data Availability

The datasets used and/or analyzed during the current study are available from the corresponding author on reasonable request.
